# Structures of Hepatitis B Virus Cores Presenting a Model Epitope and Their Complexes with Antibodies

**DOI:** 10.1016/j.jmb.2012.06.032

**Published:** 2012-10-12

**Authors:** A.M. Roseman, O. Borschukova, J.A. Berriman, S.A. Wynne, P. Pumpens, R.A. Crowther

**Affiliations:** 1 MRC Laboratory of Molecular Biology, Hills Road, Cambridge CB2 0QH, UK; 2 Faculty of Life Sciences, University of Manchester, Manchester M13 9PT, UK; 3 Latvian Biomedical Research and Study Centre, 1 Ratsupites Street, LV-1067 Riga, Latvia

**Keywords:** EM, electron microscopy, Fab, fragment of antibody, Fv, fragment of antibody variable region, HBV, hepatitis B virus, L1, linker 1, L2, linker 2, MIR, major immunodominant region, PBS, phosphate-buffered saline, 3D, three‐dimensional, cryomicroscopy, image processing, vaccine carriers, virus-like particles, improved immunogenicity

## Abstract

The core shell of hepatitis B virus is a potent immune stimulator, giving a strong neutralizing immune response to foreign epitopes inserted at the immunodominant region, located at the tips of spikes on the exterior of the shell. Here, we analyze structures of core shells with a model epitope inserted at two alternative positions in the immunodominant region. Recombinantly expressed core protein assembles into *T* = 3 and *T* = 4 icosahedral shells, and atomic coordinates are available for the *T* = 4 shell. Since the modified protein assembles predominantly into *T* = 3 shells, a quasi-atomic model of the native *T* = 3 shell was made. The spikes in this *T* = 3 structure resemble those in *T* = 4 shells crystallized from expressed protein. However, the spikes in the modified shells exhibit an altered conformation, similar to the DNA containing shells in virions. Both constructs allow full access of antibodies to the foreign epitope, DPAFR from the preS1 region of hepatitis B virus surface antigen. However, one induces a 10-fold weaker immune response when injected into mice. In this construct, the epitope is less constrained by the flanking linker regions and is positioned so that the symmetry of the shell causes pairs of epitopes to come close enough to interfere with one another. In the other construct, the epitope mimics the native epitope conformation and position. The interaction of native core shells with an antibody specific to the immunodominant epitope is compared to the constructs with an antibody against the foreign epitope. Our findings have implications for the design of vaccines based on virus-like particles.

## Graphical abstract

**Graphical Abstract:**

**Figure:**
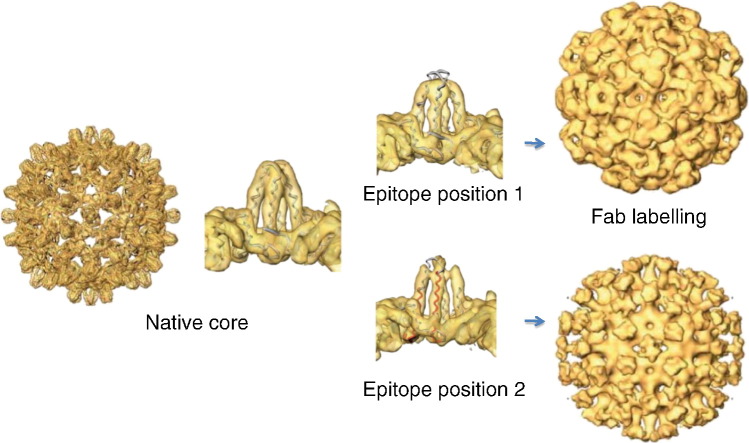

**Highlights:**

► The HBV core shell is highly immunogenic and is being used as a vaccine carrier. ► Insertion of model epitopes into the immunodominant region changes the structure. ► Alternative positions of an epitope give different structures and immunogenicity. ► The structural differences lead to different labeling with antibody fragments. ► We conclude that three‐dimensional structural analysis will be important in vaccine design.

## Introduction

The core protein of hepatitis B virus (HBV) provides an appropriate framework for display of foreign epitopes and has been suggested to provide a basis for novel vaccines.^1–3^ Core shells expressing part of the foot and mouth disease virus capsid gave protective immunity to virus challenge in guinea pigs.^4^ Clinical trials are in progress for a malaria vaccine based on a core protein displaying B- and T-cell epitopes from the circumsporozoite surface protein.^5–7^ A segment of the influenza virus M2 protein by display on core was rendered strongly immunogenic and protective in mice.^8^ It is even possible to display whole domains, for example, the 28‐kDa ectodomain of the OspA (outer surface protein A) of the Lyme disease agent, where the resulting construct conferred immunity to the agent in mice.^9^ The utility of the core shell arises in part from the special properties it has in stimulating different aspects of the immune system.^10^

**Fig. 1:**
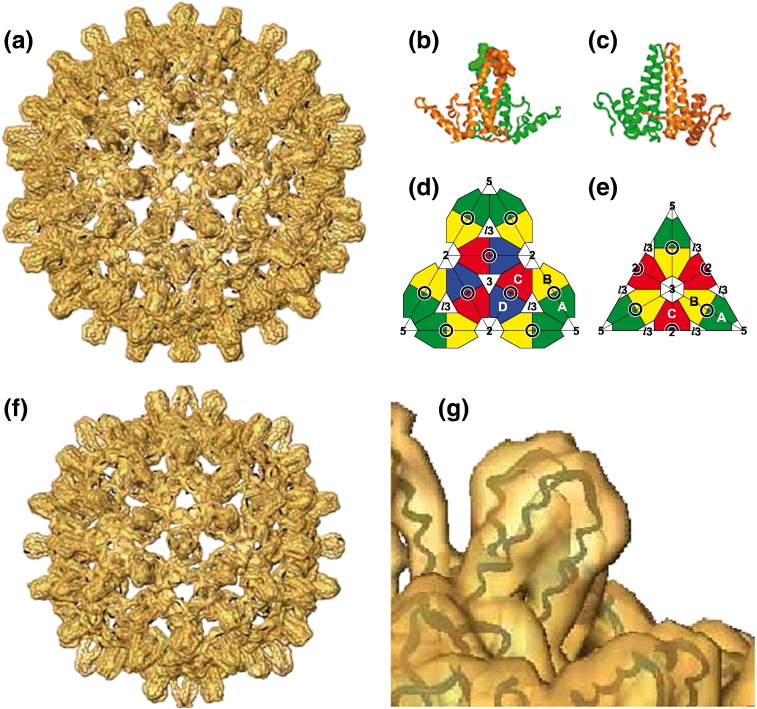
Structures of native HBV core shells. (a) *T* = 4 map from EM^12^ with the crystal structure superimposed.^18^ (b and c) Enlarged views from two directions of the dimer structure forming the spike. This is the A,B dimer (see below), which forms rings around the 5-fold axes. The A chains are closest to the 5-fold axis while the B chains face outwards from it and are closer to the 2-fold axis. The C,D spikes are positioned around the 2-fold axes. In (b), the MIR is shown in space-filling representation, as defined in Fig. 2. (d and e) Nomenclature for the structurally independent subunits A, B, C, and D in the *T* = 4 shell and A, B, and C in the *T* = 3 shell, respectively.^16^ The rings represent the positions of the dimer spikes; 2, 3, and 5 indicate the positions of the icosahedral 2-fold, 3-fold, and 5-fold axes, respectively, and *l*3 denotes the local 3-fold axes. (f) Electron microscope map of the *T* = 3 shell with dimers from the crystal structure fitted (see the text). This shell is 32 nm in diameter. (g) Detailed view of the A,B dimer of the *T* = 3 shell, showing good fit of chain from the crystal structure.

The recombinant core protein can be readily expressed in *Escherichia coli*, where it assembles into icosahedral shells of two sizes, containing 180 (*T* = 3) or 240 (*T* = 4) subunits, respectively.^11^ Hepatitis B virions contain the *T* = 4 form of the core shell.^12^ The core protein has a basic C-terminal tail that interacts with and packages nucleic acid but that is dispensable for shell formation, provided the truncation is not before amino acid 140.^13–15^ The position of the truncation influences the ratio of *T* = 3 to *T* = 4 particles.^16^ The fold of the core protein was determined by electron cryomicroscopy^17^ and an atomic structure of the *T* = 4 shell was solved by X-ray crystallography.^18^ The shells are built from dimers of core protein molecules, with each dimer producing a spike on the surface consisting of a four-helix bundle formed by apposed α-helical hairpins (Fig. 1).^17–19^ During assembly of the virion, the core undergoes a maturation step involving a relative scissor-like movement of the α-helical hairpins, which enables envelopment of the core and secretion of the virus.^12^

The core protein can remain assembly competent while accommodating foreign inserts at the N- and C-termini and more importantly at the tips of the spikes, where the immunodominant epitope of the native core protein is located. The tip itself has a large insertion capacity, accepting, for example, a 120‐amino‐acid hantavirus nucleocapsid fragment,^20^ the entire green fluorescent protein (238 aa),^21^ or a domain of the Lyme disease OspA outer protein (255 aa).^9^ For short peptides, display on the core protein greatly increases the immunogenicity of the foreign protein segment.^4,22^ Moreover, insertion in the context of the helical hairpin forming the spike is likely to constrain the conformation of an inserted peptide.^23^

The epitopes of the native core structure have been extensively investigated by using electron microscopy (EM) to examine complexes of core shells with various antibodies.^24–27^ However, no detailed structural analysis has been made of the display of foreign epitopes and their complexes with antibodies. We here describe the structures determined by electron cryomicroscopy of engineered core shells displaying at the spike tip a well-characterized epitope DPAFR from the preS1 region of the surface protein of HBV,^28,29^ against which a specific monoclonal antibody MA18/7 is available.^30^ More precise mapping revealed a DPxF sequence as a necessary and sufficient epitope for the antibody recognition.^31^ This antibody inhibits attachment of HBV to human hepatocytes^32^ and blocks model infection of primary *Tupaia* hepatocytes.^33^ Moreover, the DPAFR epitope is structurally similar to the native immunodominant DPASR/DPISR stretch at the tip of the core spike. We examined two constructs, which position the foreign epitope differently. The insertions result in an increased ratio of *T* = 3 to *T* = 4 particles. Besides showing local differences at the spike tips when compared with the native protein, the construct maps show bodily rotation of the core protein molecules, similar to those seen in maturation of the core shell,^12^ and not unexpected as the shell is known to react to conformational stress.^34^ We compared antibody complexes of the two constructs with the native structure in complex with an antibody against the immunodominant native epitope. The results demonstrate that the different insertions result in a different geometry of antibody interactions with varying degrees of steric hindrance between antibody fragments on neighboring epitopes. Understanding the changes in shell structure caused by insertions and the overall geometry of the antibody complexes may help to define the most efficient ways of displaying foreign epitopes to produce effective vaccines.

## Results and Discussion

### The native *T* = 3 core shell structure

The structure of the *T* = 4 form of the core shell is shown in Fig. 1a, with a detailed view of the α-helical hairpins forming the spike in Fig. 1b and c.^17,18^ The nomenclature^16^ for the subunits in the *T* = 4 and *T* = 3 shells is shown in Fig. 1d and e. In the *T* = 4 shell, there are A,B and C,D dimers formed by the four computationally nonequivalent subunits. In the *T* = 3 shell, the dimers are of the form A,B and C,C, with the C,C dimers lying on strict 2-fold axes.

The assembly of the core protein into *T* = 3 or *T* = 4 sized shells is influenced by truncation of the core protein at different points near the C‐terminus and by modifications elsewhere.^16,34^ Since the proportion of *T* = 3 particles increased markedly with insertions into the major immunodominant region (MIR) at the tip of the spike (Table 1), we needed a detailed model of the native *T* = 3 form of the shell, as a basis to understand the changes in the shells with insertions. Using a construct truncated at position 140, which forms mainly *T* = 3 core shells (75%), we computed a map of the native *T* = 3 shell at 8 Å resolution (Fig. 1f). We made a quasi-atomic model by docking in dimers from the *T* = 4 crystal structure,^18^ which fit well in the *T* = 3 shell (Fig. 1g).

The native *T* = 3 core particle matches the *T* = 4 particle in terms of the shape of the spikes and the shape of density around the 3-fold and 5-fold axes. The two types of *T* = 3 spikes closely resemble the *T* = 4 spikes, clearly exhibiting the bulbous knob‐shaped profile created by the crossing of the helices at the dimer interface (see Fig. 1), as opposed to the narrower conformation observed in mature *T* = 4 viral cores.^12^ The A,B dimer from the crystal structure of the *T* = 4 shell (in the immature conformation) docked precisely into the density of the spike at the local 2-fold A,B position in the *T* = 3 map. When the chains in the dimer were allowed to dock independently, there was no significant adjustment of the position or orientation of the individual subunits. At the strict 2-fold C,C position, the C-chain from the *T* = 4 crystal structure fitted best. The level of differences between the conformations of the *T* = 3 subunits themselves, and with the four different *T* = 4 solved chains, is within the degree of variability of the four chains in the *T* = 4 crystal structure.^18^

### Constructs displaying the model epitope

Two different insertion constructs are studied here, each containing the DPAFR epitope from the preS1 region of hepatitis B surface protein. These constructs, with a C-terminal truncation at position 144, were selected from a larger panel of 12 constructs that were characterized biochemically and immunologically.^29^ They are named S2-16 and S1-8. These two were chosen for structural analysis because both were expressed and formed shells relatively well, and competitive ELISA showed that the inserted epitope was fully accessible to antibody. The constructs differed in that when tested in mice, though both were immunogenic against the DPAFR epitope, the strength of the immune response induced to this epitope by the S2-16 construct was 10-fold higher than that of S1-8. The titer of the antibody response was measured by direct ELISA against preS1 peptide 21–47 containing the DPAFR epitope, which showed that dilutions of sera of 10^3^ for S1-8 but 10^4^ for S2-16 took the optical absorbance values down to background level. Background level was determined as the mean of three measurements of sera from non-immunized mice (see Table 2 of Borisova *et al.*^29^).

**Fig. 2:**
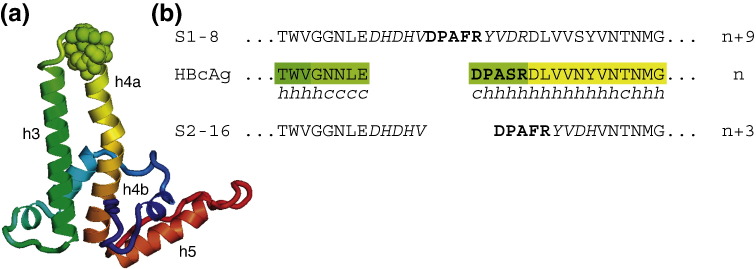
(a) Detailed structure of the subunit, shown rainbow color coded, from N-terminus (blue) to C-terminus (red). The fold that contributes to the spike consists of an outward‐going helix 3 and a returning helix 4 forming a hairpin. Helix 4 is kinked midway, giving subhelices 4a and 4b. A short loop region of 5 aa joins helix 3 to helix 4 and, together with the first few residues of helix 4a, forms the MIR (shown as space filling). This region at the tip of the spikes can be replaced with other epitopes. (b) Sequences in the region of the spike tip of the native and of the constructs with inserted epitopes. The conformation of each residue (*h* for helical, *c* for coil) in the native crystal structure^18^ is indicated beneath the native sequence. The two constructs S1-8 and S2-16 used in this study were selected from a larger panel of 12 constructs, which were characterized biochemically and immunologically.^29^ In these constructs, the native epitope (DPISR or DPASR depending on strain) has been deleted and replaced by the epitope DPAFR from the preS1 region of HBV surface protein. In the S2-16 construct, an additional 6 aa C-terminal to the native epitope were deleted. In both constructs, the loop region in the native protein following helix 3 (GGNLE) has been extended on the C-terminal side with a linker module, L1, sequence DHDHV. The DPAFR epitope follows and joins onto another linker module, L2, sequence YVDR in S1-8 and YVDH in S2-16. Hence, in both cases, the DPAFR epitope is inserted between the two linker modules, with the result that S2-16 has three and S1-8 has nine more amino acids than the native sequence.

In the native spike, the helical hairpin consists of an outward‐going helix 3 and a returning kinked helix 4, split into helix 4a and helix 4b at the kink (Fig. 2a). The immunodominant native epitope (DPASR or DPISR depending on viral strain) stands exposed on the corner of the spike at the start of helix 4a. In these two constructs, the native epitope, DPISR, has been deleted and replaced by the DPAFR epitope, inserted between two short linker sequences (Fig. 2b). On the N-terminal side, the loop following helix 3 is extended by a 5‐aa linker sequence [linker 1 (L1), DHDHV]. The DPAFR epitope follows and is joined to helix 4 by a 4‐aa linker sequence [linker 2 (L2), YVDR or YVDH]. The difference between S1-8 and S2-16 is that in S2-16, the 6 aa of helix 4a following the native epitope DPASR/DPISR have also been deleted. Thus, in these constructs, S2-16 contains three more amino acids and S1-8 contains nine more amino acids than the native sequence. In the S1‐8 construct, the foreign epitope and linker L2 might be expected to extend from helix 4a in a helical conformation, whereas in S2-16, the deletion would cause the epitope to be embedded in helix 4a.

### Comparison of the structures of the core particle constructs

**Fig. 3:**
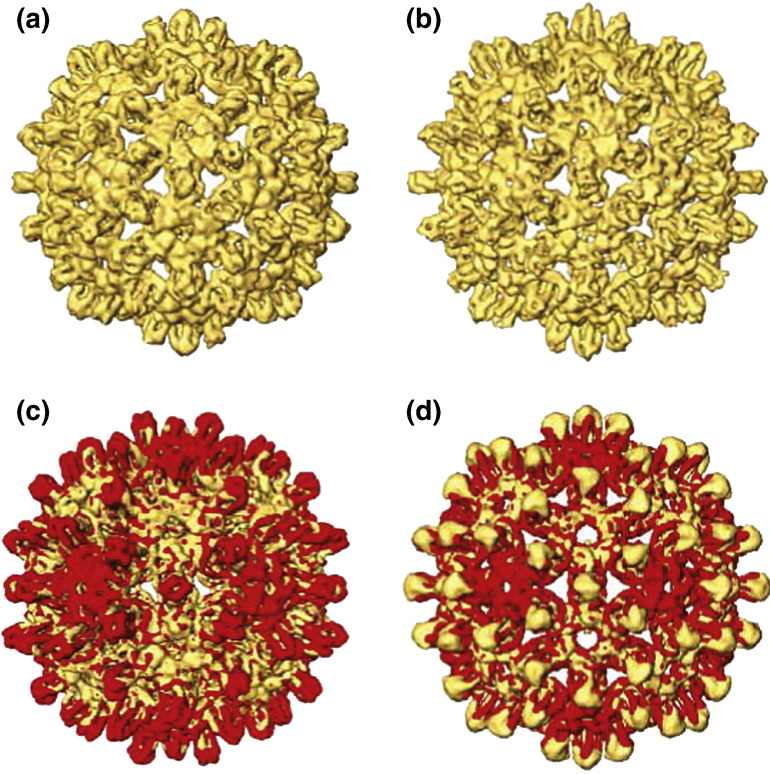
Maps of *T* = 3 core shells with inserted epitope. (a) S2-16. (b) S1-8. (c and d) The same maps in yellow with the native *T* = 3 map superimposed in red. In each case, the region of the shell surface around the 3-folds (local 6-folds) lies at the same radius as in the native construct. Around the 5-fold axes, the shell surface in the constructs has dropped, and the native shell extends to higher radius, as indicated by the red color at this position. In (c), the spike tips appear red, indicating that the spike in the S2-16 construct is shorter than that in the native, whereas in (d), the tips appear yellow, indicating that the spike in the S1-8 construct is longer than that in the native.

Images of the shells formed by the constructs showed that the particles were predominantly of the *T* = 3 size, and there was a significant number of malformed or incomplete shells (Fig. S1). Good particles were selected from the micrographs and a map of the *T* = 3 shell for each of the constructs was computed to 8 Å (Fig. 3a and b). The maps of the modified cores displaying the model epitopes look similar in overall character to the native *T* = 3 core, with well‐defined helices forming the spikes, but also show some clear differences, as outlined below.

The S1-8 map has longer spikes than the native structure, while in the S2-16 map, the spikes are shorter. When sections of the maps are viewed as densities, each spike tip has a fuzzy weak density around it, not observed in the native map, which indicates that parts of the inserted sequences may be disordered (see Fig. S2). Also, the spikes in the modified cores are slender and narrow, resembling more closely those observed in the mature *T* = 4 viral cores than those in the *T* = 3 or *T* = 4 cores of the recombinantly expressed protein.^12^ The density in the shell region (excluding the spikes) agrees closely in most places, except that around the 5-fold axes, the shell region of the construct maps has dropped to a lower radius (Fig. 3c and d). The changes in conformation can be accounted for largely by a bodily rotation of the subunit, but some internal changes are also needed to fully explain the differences.

**Fig. 4:**
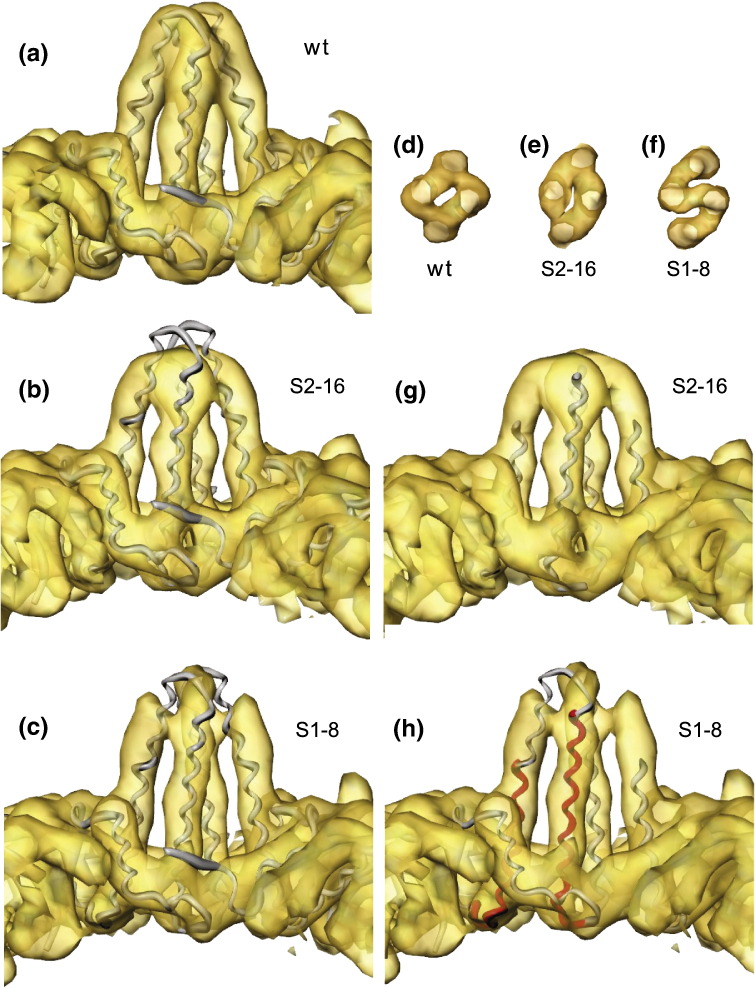
Spike conformations in the modified cores compared with the native type (wt). (a) Spike at the strict 2-fold symmetry axis showing the native *T* = 3 map and atomic model. (b) Map of the S2-16 construct overlaid on the native *T* = 3 model spike, same view as in (a). (c) Map of the S1-8 construct overlaid on the native *T* = 3 model spike, same view as in (a) and (b). (d–f) Radial views of the tip of the spike, showing native (d), S2-16 (e), and S1-8 (f). In each case, the C,C dimer is shown, but the computationally independent A,B dimers in each construct display very similar features. In particular, the additional bridging density seen in S1-8 (f) compared with wt (d) or S2-16 (e) is thus unlikely to have arisen from the averaging of noise. (g) S2-16 spike, same view as in (a) to (c), with molecular fragment of spike, as shown, reoriented to fit in the density. (h) S1-8 spike, same view as in (a) to (c), and (g), with fragment of spike reoriented to fit in the density. The fitted fragment is shown in red and is the same fragment that was fitted in (g). The larger fragment in gray has been aligned with the red fragment to show the improved fitting in the extended part of helix 4a as well as the loop region. The fragment fitted included helix 3 and helix 4b, but not helix 4a or the loop region between helix 3 and helix 4a. One turn of α-helix before helix 3 and beyond helix 4b was also included. (b) and (c) show the mismatch of the native structure to the constructs. (g) and (h) show how the change can be modeled largely as a rigid‐body movement of the subunits. (f) shows the bridge of density joining the subunits in S1-8. (e) shows a distortion compared to (d), but not the bridge shown in (f).

When the atomic model of the *T* = 3 core, obtained by fitting the subunits to the native *T* = 3 map (Fig. 4a), is overlaid on the construct maps (Fig. 4b and c), the hairpin helices forming the spike do not lie correctly in the density of either construct map. It is apparent that the subunits need to be tilted to fit the helices into the map density (Fig. 4g and h), which can be achieved quantitatively by docking of the native core protein chains as individual subunits into the construct maps. The change in conformation in the shell can be accounted for mostly by a rigid‐body rotation of the subunits by a few degrees about an axis roughly perpendicular to the plane of the interface between the subunits of the dimer. The effect is to reduce the crossover of the helical hairpins from apposing dimers, resulting in a narrower profile of the spikes, much like that observed in the *T* = 4 cores obtained from mature virions.^12^ The rotation (of ~ 6°) is largest for the A chains, which are located in rings around each 5-fold axis. These C-terminal helix 5s (Fig. 1b, c, e, and f) associate in a ring around the 5-fold axis, forming the shell layer at this position. The rigid‐body rotation of the subunit, bringing the spike helices into a more radial orientation, also has the effect of pivoting the helix 5s about a point near the base of the helices forming the hairpin, causing the shell layer around the 5-fold axis to drop to a lower radius (Fig. 3c and d). The subunit at the B position rotates less and pivots about the C-terminal tip of helix 5, leaving the shell radius unchanged here. The subunits forming the spike at the C positions also show a small rotation, also pivoting about the C-terminal tips of helix 5, and achieving the more slender spike profile.

It is clear that though the fit of the subunits into the spike density of the constructs has improved through rigid‐body reorientation of the subunits, smaller additional changes are also needed to explain the observed differences. In particular, the outer segment of kinked helix 4 (helix 4a) needs to be reoriented into density, predominantly as a rigid body, but the exact position of the tip of helix 4a cannot be certain from interpreting maps at this resolution, though the strong density associated with the spike structure is consistent with a helical structure. The degree of tilt of helix 4a needed to fit it into the density in the outer region of the spike is ~ 20° for S2-16 and ~ 10° for S1-8. Since α-helices are resolved as strong tubular densities in these maps at 8 Å resolution, the helices in the constructs can be compared with those in the native core, and their lengths can be estimated with an accuracy of one turn of the α-helix (~ 3 or 4 aa). It is clear that helix 3 and helix 4a in the constructs show differences in length compared with those in the native core. This affects the position of the foreign epitope in the constructs, as will now be described.

### Positioning of the foreign epitope

The S2-16 spike appears to be a shortened version of the native one (Fig. 4b and g). Helix 3 looks the same length but helix 4a is shorter by ~ 1 turn (Fig. 4b). (In the diagrams shown in Fig. 4, helix 4 of the core protein subunit appears on the left and helix 3 appears on the right in the hairpin nearer to the observer.) This is consistent with the fact that 2 aa have been removed from within this helix and suggests that the inserted linker sequence L2 must in this case be mostly helical. The connection between the unchanged helix 3 and the shortened helix 4 appears the same as in the native; hence, the 5‐aa linker L1 added to the loop region does not form a defined density and is not seen. It probably gives rise to the fuzzy weaker density apparent around the spikes. In summary, in S2-16, L1 is disordered, and L2 has been incorporated within helix 4 to replace the deleted helical part of the native structure. The DPAFR epitope is likely to adopt a helical configuration to replace the native DPASR/DPISR epitope on the end of the shortened helix 4a.

In the map of the S1-8 construct, helix 4a also appears about one turn shorter at its N-terminal end compared with helix 4a in the native structure (Fig. 4c and h), indicating that in this construct, the inserted linker sequence L2 does not continue the α-helix. Helix 3 appears to be extended by one turn. The DPAFR epitope is positioned between the two linkers L1 and L2. Since the S1-8 spike is longer than the native spike, the linkers must extend outwards from their constrained end points and present the epitope at the tip of the extended spike. This is a quite different configuration to the native epitope or to the foreign epitope in S2-16. The strong additional feature in S1-8 at the tip of the spike lying across the 2-fold axis (Fig. 4f *versus* Fig. 4d and e) indicates extra material and a new interaction between the subunits at the dimer interface. This compact density may in part correspond to the inserted DPAFR epitope forming a short helix. In the solution structure of the preS1 protein from the HBV surface antigen, a short region containing part of the DPAFR epitope was shown to be helical.^35^ The fuzzy density surrounding the spike indicates that there is some disordered chain in this structure too (Fig. S2).

Thus, the sequence modifications at the tips of the spikes cause entire subunits to undergo similar rigid‐body rotations, giving rise to the similar observed changes in the shell region of the structure. The overall rigid‐body rotation of the subunits is also responsible for the changes in the profile of the spikes, making them more slender than in the native shell. In addition, helix 4a reorients, causing or caused by a reconfiguration of the tip structure, which is related to the overall subunit reorientation. In both constructs, some of the inserted linker sequence is disordered. The structure of S1-8 was not as expected, since the inserted linker sequences caused helix 3 to be extended rather than helix 4a. Together with the additional partially ordered material, this makes the S1-8 spikes longer than those in the native structure. In the S2-16 construct, the shortening of helix 4a and the disordering of the loop at the tip make the spikes look shorter than in the native structure.

### Complexes of Fabs with the native core shell

An initial study of a complex of the well‐characterized C1-5 antibody,^36–38^ which recognizes the immunodominant DPISR epitope (more precisely 78-DPIxxD-83)^38^ at the tips of the spikes of the native core shell, was made to allow comparison with the interactions of the MA18/7 antibody with the engineered core shells. Particles were mainly (90%) of the larger *T* = 4 form, and a map of the *T* = 4 complex computed to 8 Å resolution gives an indication of the level of detail that can be obtained in a favorable case. As this complex has already been studied,^27^ only brief details are given here to allow comparison with the constructs with a foreign epitope.

**Fig. 5:**
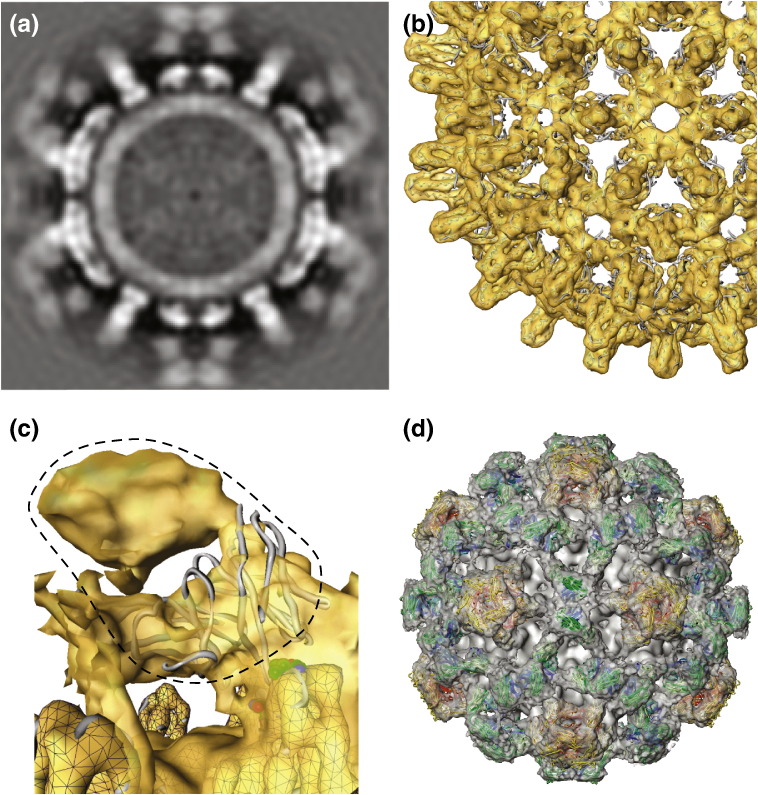
Map of the complex of C1-5 Fab with the native *T* = 4 shell. (a) Central slice through Fab complex map. (b) Core protein region of antibody complex. The Fab density is weaker than the core [see (a)], because of low occupancy of sites due to blocking, and has been suppressed by setting the display surface at a high level. The core density is sharply defined and the coordinates from the crystal structure (overlaid gray tubes) fit precisely. (c) Close‐up view of one spike and its associated Fab density (outlined with the broken line) with the modeled Fv chain (drawn as a gray tube) docked into the density adjacent to the epitope (shown as colored space filling). The density more distant from the epitope corresponds to the constant domain of the Fab. The surface of the core protein [as in (b)] is indicated by mesh work. (d) Map of the whole complex with Fabs modeled in to show how the overall density arises, although not all sites can be occupied because of blocking (see the text; Fig. S4). For clarity, the model represents 100% occupancy of A and C sites, with no labeling on B or D sites. Fabs on A sites have their heavy and light chains colored red or yellow, respectively. Those on C sites are colored green (heavy chain) and blue (light chain). Since the occupancy of the B sites is very low, and there is a large overlap of the Fabs on the C and D sites, the modeled Fabs fill most of the observed EM density. Figure S3 shows a section similar to (a), with annotation delimiting the shell region from bound Fabs. Map of the complex of C1-5 Fab with the native *T* = 4 shell. (a) Central slice through Fab complex map. (b) Core protein region of antibody complex. The Fab density is weaker than the core [see (a)], because of low occupancy of sites due to blocking, and has been suppressed by setting the display surface at a high level. The core density is sharply defined and the coordinates from the crystal structure (overlaid gray tubes) fit precisely. (c) Close‐up view of one spike and its associated Fab density (outlined with the broken line) with the modeled Fv chain (drawn as a gray tube) docked into the density adjacent to the epitope (shown as colored space filling). The density more distant from the epitope corresponds to the constant domain of the Fab. The surface of the core protein [as in (b)] is indicated by mesh work. (d) Map of the whole complex with Fabs modeled in to show how the overall density arises, although not all sites can be occupied because of blocking (see the text; Fig. S4). For clarity, the model represents 100% occupancy of A and C sites, with no labeling on B or D sites. Fabs on A sites have their heavy and light chains colored red or yellow, respectively. Those on C sites are colored green (heavy chain) and blue (light chain). Since the occupancy of the B sites is very low, and there is a large overlap of the Fabs on the C and D sites, the modeled Fabs fill most of the observed EM density. Figure S3 shows a section similar to (a), with annotation delimiting the shell region from bound Fabs.

The map of the complex (Fig. 5a) shows that the diameter of the particle has increased from 350 Å for the unlabeled core to 470 Å in the complex, illustrating the large size of the antibody compared to the spikes on core shell (see also Fig. S3). Because the density for the fragment of antibodies (Fabs) is weaker than that for the core (see below), the core itself in the complex can be visualized by choosing a density threshold intermediate between Fab and core (Fig. 5b). The structure thus displayed closely matches the uncomplexed core (Fig. 1a), as shown by the good fit of the crystal structure into the density (Fig. 5b). Comparison of the map of the complex displayed at a lower density level (Fig. 5d) with the view of the core shell (Fig. 5b) shows that the Fabs form an additional outer shell of density with two main motifs, a turret-like nearly cylindrical density on the 5-fold axis and a rectangular bridge of density across the 2-fold (local 6-fold) axis. These features represent superpositions of antibody fragments bound to a subset of the icosahedrally related sites. The computed map is icosahedrally averaged, and at each position, the density represents the average of the occupancy over all the symmetrically related sites.

Examination of the additional density due to the Fabs indicates that the four independent binding positions on the A, B, C, and D subunits have differing occupancies, demonstrating that their environments are distinct from each other. Antibody binding at one site may block binding at another site, as has been reported previously, for example.^39^ In the core protein, the spike, being a dimer, contains two closely positioned quasi-equivalent antibody binding sites. The footprint of binding of the Fab to one site overlaps with the other; thus, only one of these can be occupied at once, giving a maximum averaged occupancy level of 50%, for all potential sites (Fig. S4). The Fabs project off the epitope at the corner of the spike at an angle of about 45° to the radius (Fig. 5c), as observed previously.^27^ Because of this orientation, the Fab fragments extend out sideways over a relatively long distance and block access to binding sites on neighboring spikes. The A chains are arranged around the 5-fold axis, and their bound Fabs face inwards towards the 5-fold axis, forming the turret-like feature. The average occupancy for the A site is 0.4, indicating that, on average, two of the possible five A sites are filled. In the icosahedrally averaged map, this is represented as a partial occupancy of 0.4 at all the sites, and the density at the turret-like feature is an average of 2 Fabs per 5-fold motif, bound at random positions. Molecular modeling (Fig. 5c and d and Fig. S4) indicates that a maximum of two of these sites (of five potential A sites) can be filled at once, in good agreement with the measured occupancy.

Similar effects cause the partial occupancy around the 2-fold (local 6-fold) axes, where the three different types of quasi-symmetry‐related sites (on the B, C, or D chains), six in total, can clash, giving rise to the bridge-like features. The occupancy at the B site, B being the outward facing chain of the dimers arranged around the 5-fold axis, was measured as less than 0.1. This low occupancy can be explained by the blocking effect of Fabs at the C and D sites. The occupancy on the C and D chains positioned around the local 2-fold axis was 0.4 and 0.2, respectively. The difference in occupancy at these two similar sites could be due to their different structures or environments—which is a consequence of the quasi-symmetry. The orientation of Fab binding and separation of spikes on the core appear to be compatible with bivalent binding of an antibody.

### Fab complexes with the modified core shells containing inserted epitopes

**Fig. 6:**
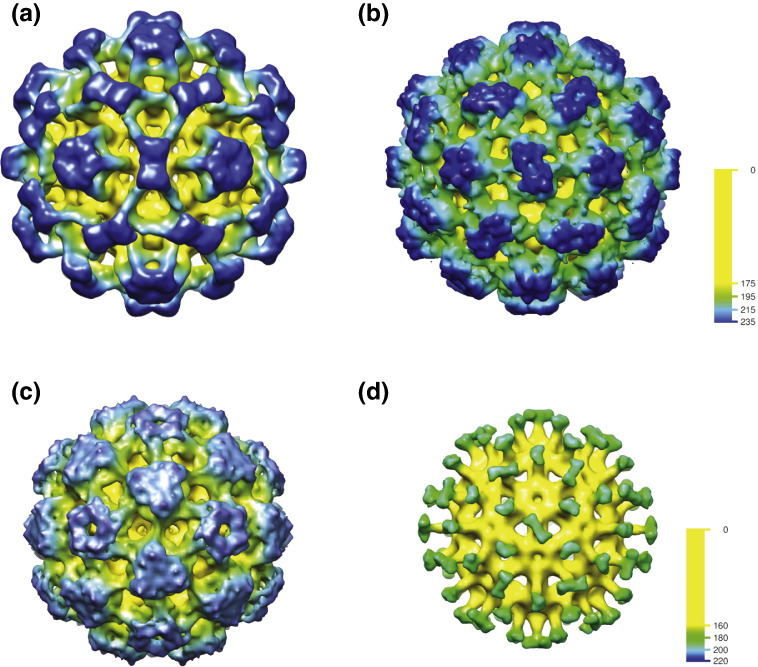
Maps of various complexes of Fabs and core shells. (a) *T* = 4 native shell with C1-5 Fab (as Fig. 5a and d, but at lower resolution to compare with construct maps). (b) *T* = 4 S2-16 construct with MA18/7 Fab. (c) *T* = 3 S2-16 construct with MA18/7 Fab. (d) *T* = 3 S1-8 construct with MA18/7 Fab. Note that the maps in (a), (b), and (c) have a similar character, with the Fabs coming together to make strong features around the symmetry positions [5-fold and 2-fold in (a) and (b); 5-fold and 3-fold in (c)], whereas in (d), the density arising from the Fab is radial and does not create features around the symmetry axes. The maps were filtered to 15 Å resolution. The radial color scales shown are calibrated in angstroms (Å). The core shell is colored yellow, and then the color graduates in 20‐Å bands from green, to light blue, to dark blue as the radius increases. The outer limit of the shell radius is 160 Å or 175 Å for the *T* = 3 or *T* = 4 shells, respectively.

The core shell constructs were labeled with Fabs derived from the monoclonal antibody MA18/7 to the DPAFR epitope. The maps of the labeled S2-16 complex are similar in character to the C1-5‐labeled native complex. Figure 6a and b show equivalent views of *T* = 4 maps of the native and S2-16 Fab complexes, at the same nominal resolution of 15 Å. The Fabs project out from the spikes at a similar angle to form similar motifs at the 5-fold or 2-fold symmetry axes. Slight orientation differences could be due to the different nature of the interaction in the two complexes, as well as the epitope being presented differently. In addition, it is possible that the two different antibodies (C1-5 and MA 18/7) exhibit slightly different elbow angles.^40^ However, the major axis of corresponding Fabs lies in a very similar direction and thus gives rise to very similar maps. Since they are so similar, it is very likely that the position and orientation of presentation of the epitopes are very similar and that the model epitope has essentially replaced the native epitope at the top of helix 4.

Cross-reactivity of the antibodies has been tested in Western blot, direct and competitive ELISA, and immunogold EM (P.P. *et al.*, unpublished results). The anti-DPAFR MA18/7 antibody does not cross-react with the native DPASR or DPISR core epitope in Western blot, ELISA, or immunogold EM. No sign of even weak reaction was ever seen. Vice versa, the C1-5 antibody does not recognize S1-8, S2-16, other HBc (core protein from HBV) VLP carriers bearing the DPAFR epitope and lacking native DPISRD sequence, or RNA phage coat protein-preS1 fusions, which contain DPAFR epitope in different amino acid neighborhood (P.P. *et al.*, unpublished results).

Comparison of the *T* = 4 and *T* = 3 S2-16 maps (Fig. 6b and c), which have identical Fab binding at the molecular level, illustrates how the same features manifest differently according to the different *T* = 3 or *T* = 4 packing. The turret‐like motifs around the 5-fold axes correspond closely, but in the *T* = 3 structure, the bridge across the local 6-fold axis has strict 3-fold symmetry, as opposed to having strict 2-fold symmetry in the *T* = 4 structure. Apart from the symmetry difference, these bridges are similar in character.

Since the majority of the S1-8 particles were of the *T* = 3 type, only a *T* = 3 map of the S1-8 Fab complex was calculated. In Fig. 6c and d, the S2-16 *T* = 3 map is compared with the S1-8 *T* = 3 map. The additional density due to the Fabs in the S1-8 map extends radially outwards from the spikes on the shell, rather than at an angle (~ 45°) as for the S2-16:MA18/7 Fab or native core:C1-5 Fab complexes. The S1-8:MA18/7 Fab map does not show the same motifs as the native core:C1-5 Fab or S2-16:MA18/7 Fab maps on the 5-fold or 3-fold axes. Because of the radial disposition of the Fabs in the S1-8 complex, the density due to Fabs is less distinct than for the S2-16 complex, as it does not form the distinctive “bridges” or interacting motifs across the symmetry axes. Thus, blocking between sites on adjacent spikes does not occur, and the blocking effect is restricted to the overlap of the two closely spaced sites on the tip of a given spike.

The Fab density on S1-8 is strongest near the tips of the spikes on the core shell. It becomes weaker with increasing distance from the surface, indicating a level of flexibility or disorder. The density lying close to the dimer interface (2-fold axis for C,C spike, or local 2-fold for A,B spike) is enhanced by 2-fold averaging (computational 2-fold averaging at the C,C spike, or superposition of quasi equivalent densities on the A,B spike). This combination of disorder and 2-fold averaging makes it difficult to interpret the outer density in detailed terms of the shape of a Fab.

An additional density feature observed at the tip of the spike in the S1-8 unlabeled core (Fig. 4f) indicates that the DPAFR epitope lies close to the 2-fold or local 2-fold axes at the tip of the spike of the dimer in the S1-8 construct. This explains why the DPAFR epitope on S1-8 is accessible, though in a very different environment to S2-16. It also raises the possibility of interference of the two epitopes on the dimer with one another, since they are in close proximity. The location of the epitope in an unconstrained position in a loop at the tip of the spike may also explain why in this complex the Fab fragments do not appear well ordered. Since S1-8 and S2-16 contain exactly the same epitope, the different levels of immunogenicity observed in mice must be due to the way it is presented.

In summary, in S2-16, the model epitope is in a position and conformation closely resembling the immunodominant epitope in the native shell. Bound Fabs project from the “corner” of the spike, at an angle of ~ 45°, causing blocking effects between sites on different spikes. In S1-8, the DPAFR epitope is located more centrally on the tip of the spikes, near the dimer interface and the 2-fold axis. The density of bound Fabs projects radially outwards from the center of the spike. No blocking between sites on different spikes will occur with this geometry. The footprint of antibody binding will only allow one antibody or Fab to bind per spike on either of the constructs or to the MIR on native cores.

## Conclusions

HBV core protein has an inherent and complex immune stimulating capability,^10^ which is both T-cell independent and T-cell dependent.^41^ The native core protein assembled into particles can present many copies of an epitope with high density and appropriate spacing to cross-link B‐cell receptors optimally.^42^ For the native particle, this produces a strong antibody response against the major immunodominant epitope positioned at the tips of the spikes on the core shells. By removing the native immunodominant epitope and inserting a foreign sequence in its place, it is possible to elicit a strong immune response to the inserted sequence. The inserted sequence may be a large protein domain or just a small peptide. In the latter case, as we have studied here, the exact positioning of the foreign sequence can have large effects on the immunogenicity of the construct.

For the modified core construct to induce an effective immune response, the displayed foreign epitope must be able to bind strongly to receptors on B-cells in a way that promotes the cross-linking of clustered receptors. The structure of the MIR at the tips of the HBV core protein spikes potentially allows the inserted epitope to be either displayed as a loop constrained at both ends by fixed α-helices in the hairpin or incorporated in a helical conformation at the end of one of the radially extending helices. The two constructs studied here appear to exemplify these two modes of display. Small changes in the construct, for example, in the number of amino acids in the insert, can have large effects on the orientation and accessibility of the epitope. If the epitope is embedded in an α-helix, then a change of one amino acid position in the sequence can cause the epitope to be reoriented by 100°. In addition, changes made to the core protein around the MIR cause a change in orientation of the subunits in the dimer forming the spikes on the exterior of the core shell and this may affect the orientation and accessibility of the epitope presented.

In the S2-16 construct studied here, the inserted foreign epitope appears to be positioned in a partly helical conformation that closely mimics that of the native immunodominant core epitope. The binding of antibodies C1-5 and MA18/7, respectively, to the two kinds of shell is very similar, with the Fab fragments projecting off the “corners” of the spikes at an angle of ~ 45° to the radius. This overall arrangement is therefore likely to be a favorable one for strong interactions between core shell and membrane‐bound B‐cell receptors, exploiting an equivalent geometry. Like the native core protein, S2-16 might therefore be expected to produce a strong immune response, as indeed it does.^29^ In the S1-8 construct, by contrast, the foreign epitope appears to be displayed as a less ordered feature right at the tip of the spike and the bound Fab fragment lies in a radial direction. This Fab arrangement may indicate a much less favorable geometry for interaction of the foreign epitope with the equivalent arm of a membrane‐bound B‐cell receptor, leading to less efficient cross-linking and a correspondingly weaker immune response than that observed against S2-16.^29^ In addition, in S1-8, the epitope is presented exposed near the 2-fold axis right at the tip of the dimer spike. This might allow interactions with the partner epitope from the other member of the dimer to change the molecular surface presented. In the S2-16 construct, this could not occur. There is also the question of whether a small inserted sequence in the engineered core adopts a conformation like that of the epitope in the context of its native protein. In our case, monoclonal antibody MA18/7 was raised against native surface antigen,^30^ and since both S1-8 and S2-16 react with MA18/7 and, in mice, induce production of antibodies that react with the native preS1, they must both be displaying the DPAFR epitope in something close to its native conformation. However, in S2-16, the epitope is embedded in the native-like α‐helix 4a, which may have a stabilizing effect on its conformation. This more rigid and defined location and environment may also favor stronger interactions with the membrane‐bound B‐cell receptors. The organization of the linkers in S1-8 does not provide the same level of constraint. Different modes of insertion may have different constraints, and it may be that the SplitCore approach,^43^ which provides a less constrained setting, will prove advantageous especially for larger domain inserts.

Therefore, in the design of virus-like particles for use as vaccine carriers, it is important to consider the three‐dimensional (3D) structure of the entire particle. Since core particles carrying preS1 epitopes are considered as a prospective therapeutic vaccine against chronic hepatitis B,^2^ our data may help in this particular case. The overall conformation changes in the spikes observed for the two different constructs were similar; hence, this information could be used when designing constructs with other epitopes. However, especially since the core particles are not strictly rigid scaffolds to which the epitopes become attached and it is also hard to predict the conformation that will be adopted by any particular inserted sequence, it will help to visualize the structures of further constructs and relate them to the results of functional assays.

## Materials and Methods

### Production of core shells

Cloning, expression, and purification of the HBV core particles S1-8 and S2-16 with DPAFR epitope inserted at the MIR were performed as previously described.^29^ Briefly, *E. coli* K802 (*hsdR*, *gal*, *met*, *supE*, *mcrA*, *mcrB*) cells were grown overnight on a rotary shaker at 37 °C in 750‐ml flasks containing 300 ml of M9 minimal medium supplemented with 1% casamino acids (Difco Laboratories, USA) and 0.2% glucose. An OD_540_ (optical density at 540 nm) of 2–5 was usually reached. Cells were pelleted and lysed by 30‐min incubation on ice in lysis buffer containing 50 mM Tris–HCl, pH 8.0, 5 mM ethylenediaminetetraacetic acid, 50 μg/ml PMSF, and 2 mg/ml lysozyme, and then ultrasonicated 3 times for 15 s at 22 kHz. Lysates were adjusted to 10 mM MgCl_2_ and 20 μg/ml DNase and after 5‐min incubation to 0.1 M urea. After low‐speed centrifugation, proteins were precipitated from the supernatant with ammonium sulfate at 33% saturation for 1–2 h at 4 °C. Ammonium sulfate precipitates were washed with phosphate‐buffered saline (PBS) buffer and then dissolved in PBS containing 1.5 M urea and 0.6% Triton X-100 just before loading onto the Sepharose CL4B column (2.5 cm × 85 cm). The S1-8 and S2-16 capsids were eluted from the column with PBS buffer containing 0.25 M urea and 0.01 % Triton X-100. The presence of S1-8 and S2-16 polypeptides in fractions was tested by PAGE. Positive fractions were pooled and concentrated by ammonium sulfate precipitation at 33% saturation for 20 h at 4 °C. Pellets were resuspended in PBS or in Tris–saline buffer—10 mM Tris–HCl, pH 7.5, and 150 mM NaCl—to a final concentration of about 2 mg/ml, dialyzed overnight against 2000 volumes of the same buffer, and stored at − 70 °C in 50% glycerol.

The native *T* = 4 particles for labeling with the C1-5 antibody were prepared exactly as described previously, for the full‐length CW isolate.^12^ For isolation of *T* = 3 particles, the construct HBcΔ-CW, in which the core protein is truncated after amino acid 149,^44^ was subject to a further truncation after amino acid 140. This was produced by PCR using oligonucleotide primers 5′ GA CTT CAA CAT ATG ACA TTG ATC CTT ATA AAG 3′ and 5′ CCG GAA TTC TCA TAA GAT AGG GGC ATT TGG 3′. The resulting PCR product was cleaved with NdeI and EcoRI and inserted into the corresponding restriction sites in the expression plasmid PT7-SC. Preparation of *T* = 3 shells was then done in the same way as for *T* = 4 shells,^44^ but with the modification that in the final sucrose gradient centrifugation step, the later fractions of the peak were selected.

### Antibody labeling

Fabs from the C1-5^36^ or MA18/7^30^ antibodies were prepared by digestion with Papain,^45^ using Pierce Kit 44885 (Thermo Fisher Scientific, Cramlington, UK). Antibody fragments were mixed with core shells, at a ratio of ~ 1 Fab per HBV core dimer, before freezing on EM grids.

### Electron cryomicroscopy

Samples of core shells, or antibody complexes, were prepared for EM on holey carbon films, by standard methods.^12,17^ Frozen hydrated samples were imaged at 300 kV or 200 kV, with a nominal magnification of 50,000 ×, on an FEI Tecnai F30, or F20, or 60,000 × on a Hitachi HF-2000 electron microscope. Images were recorded on Kodak SO-163 film and developed in full‐strength Kodak D19 for 12 min. Films were digitized using a Zeiss SCAI Scanner (Z/I imaging, Oberkochen), sampling at 7 μm per pixel. Two-by-two pixel binning was then applied, giving a resultant 14 μm per pixel. Table S1 shows a summary of imaging conditions and parameters for each structure analyzed.

### Image analysis and 3D reconstruction

Image processing, 3D map computation, and refinement of the magnification were performed as before,^12^ using the MRC programs for icosahedral reconstruction.^46,47^ Particles were selected manually using Ximdisp^48^ or automatically using FindEM.^49^ The sampling for the images of the non-native‐labeled particles was reduced by a further factor of 2 by binning adjacent pixels. SPIDER^50^ procedures were used for iterative alignment and centring of particles. Imagic^51^ was used for multivariate statistical analysis and classification of the aligned particle sets. A significant portion of the non-native core particles suffered from poor assembly. Classes of particles forming complete, nondeformed shells were selected visually. These classes appeared unbroken and isometric. The common‐lines procedure^47^ was used to find the orientation of the particles directly from the class averages. The four or five best scoring class averages were used to reconstruct an initial 3D model. After this, individual particles from the selected classes were refined iteratively against projections of the current best 3D model, as before.^11,12,17^ Initially, the defocus used to fit a contrast transfer function for each image was determined from the summed power spectra of the particles.^17^ The contrast transfer function was refined iteratively in the final stages of the reconstruction procedure.^12^ Finally, the maps of unlabeled cores were sharpened by weighting the Fourier amplitudes so as to match the rotationally averaged one-dimensional amplitude profile with the atomic model of the *T* = 3 shell (as fitted), as previously described.^12^ Maps of unlabeled cores were reliably determined to 8 Å resolution (Fig. S5), as defined by the Fourier shell correlation criterion 0.33.^52^ The maps of Fab labeled cores were determined to beyond 15 Å, or 8 Å in the case of the native core:C1-5 Fab complex (Figs. S6 and S7. The final maps of unlabeled cores and the native core:C1-5 Fab complex were limited to 8 Å resolution by Fourier truncation for analysis, comparison, and display. The Fab complexes of S2-6 and S1-8 were similarly limited to 15 Å for analysis and display. The hand of the native *T* = 3 and *T* = 4 maps was assigned directly based on the distinctive handed motifs seen at the 5-fold axis (since the atomic model for *T* = 4 was available). The hand of the other, lower‐resolution, maps could be assigned based on comparing coarser handed features with the known *T* = 3 and *T* = 4 maps.

### Docking and modeling

DockEM^53^ was used to fit models and fragments into 3D maps. A magnification search with atomic models and the EM maps was used to determine the magnification of the EM maps accurately. A modification of DockEM, to restrict searches to rotations about a specified pivot point, was implemented for fitting of helix fragments. *T = 3 quasi-atomic model:* The chains from the crystal structure of the *T* = 4 shell^18^ were fitted as dimers, and as individual chains, into all three unique positions in the *T* = 3 map, using DockEM. For docking, a density was created from the atomic models and filtered to match the resolution of the EM map, 8 Å. Once a preliminary fit of the dimer had been obtained, the relative magnification was optimized by including a relative scale in the local search to match the dimer in the density.

A full atomic model of the *T* = 3 shell was created by applying icosahedral symmetry to the fitted chains at the A,B and C,C spikes, using the CCP4^54^ program PDBSET. A density map created by filtering this model to 8 Å was used to optimize the magnification scale of the other *T* = 3 core shell maps, by correlation in DockEM. A scale search repeated using the native *T* = 3 EM map, or a density model from the coordinates created with the tips of the spikes deleted, produced the same results. *Modeling of core particles with DPAFR epitope inserted:* Superposition of the maps indicated changes in shell region and tips of spikes. Superposition of the native *T* = 3 model and maps of the modified core particles also demonstrated changes. Initially, changes at the tip were not modeled due to the weaker less defined density in this region. A series of fragments of the subunit, excluding tip region, were created and docked into the density. It was found that changes in the shell domain could be modeled as rigid‐body reorientations of the subunit structure. The tip changes were modeled by docking of small helical segment representing helix 4a to the existing fitted model. The extension of helix 3 in the S1-8 map was examined by superpositioning a model helix to extend from the tip of helix 3. *Fragment of antibody variable domain (Fv) docking and occupancy analysis:* An atomic model of the core particle antibody complex was obtained by docking a model of the Fv of antibody C1-5 into the map density, using information to 8 Å resolution. The docking of the Fv at the density associated with chain C proved the most reliable, due to a combination higher occupancy and less superposition effect than the A site. Symmetry‐related positions on the other three chains were obtained by applying the quasi-symmetric relationships of the underlying chains. This enabled a full atomic model of the core shell Fv complex to be obtained. The atomic model of the C1-5 Fv was obtained by submitting the sequence^37^ to the Web Antibody Modeling server.^55^

A model of the core shell with complete Fabs bound was obtained using an antibody model chosen to have a compatible elbow angle^40^ with the density observed, selected from the Protein Data Bank (Protein Data Bank code: 1DBA). The Fv region of this model was aligned to the positioned C1-5 Fv models. The occupancy levels at all the different sites were determined by creating a simulated density from the atomic model of this complex, for all permutations of occupancy, in steps of 10%. The simulated densities were correlated with the observed EM map of the complex using DockEM,^53^ using information to 15 Å resolution, with the best match indicating the correct occupancy. On the scale used, the maximum possible occupancy per binding site on each chain is 1. However, because only 1 of the 2 sites per dimer can be occupied, the maximum possible occupancy for C + D or A + B is 1; that is, if there was 100% C site occupancy, then no D sites could label. Therefore, if the A,B and C,D spikes were 100% labeled (i.e. one Fab per spike), then the total occupancy of all the sites would add up to 2. Atomic coordinate alignments and transformations were computed using Amira Software (Visage Imaging GmbH, Berlin, Germany), as were map rendering and molecular representations for Figs. 1–5. Figure 6 was made using UCSF Chimera.^56^

## Figures and Tables

**Table 1:** Percentage of shells (*T* = 3 or *T* = 4) for various constructs

	CW full length	CW trunc140	Riga full length^a^	Riga trunc144^a^	S2-16 trunc144	S1-8 trunc144
*T* = 3	10	75	10	20	60	80
*T* = 4	90	25	90	80	40	20

a From Borisova *et al*.^29^
