# Supplementary material for: Structures of Hepatitis B Virus Cores Presenting a Model Epitope and Their Complexes with Antibodies
Source: J Mol Biol. 2012 Oct 12;423(1):63–78. doi: 10.1016/j.jmb.2012.06.032 (PMC3465560; doi:10.1016/j.jmb.2012.06.032)
Supplement: Supplementary file 1 — Supplementary materials [file mmc1.pdf]

Supplemental Figures

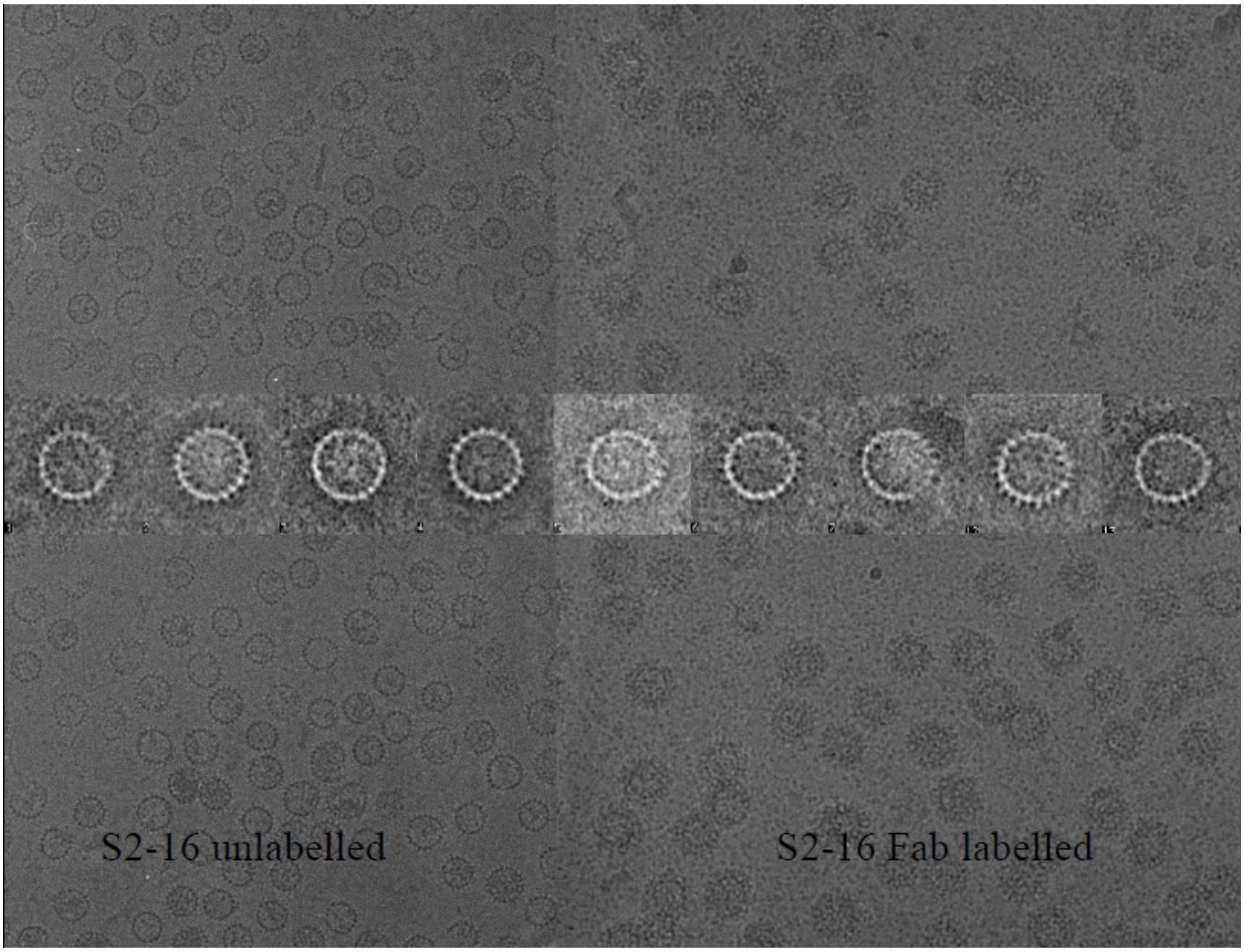

Figure S1. CryoEM images (left) and class averages (central panel) of the unlabelled core particles of the S2-16 construct. Fab labelled core particles are shown on the right. The class averages (enlarged, with inverted contrast) show that there is some distortion of the modified core shells. Boxed images were aligned using Spider[50] procedures, and classified using MSA procedures in Imagic[51]. In the individual images it is not possible to assess the quality of the shells. Only images from spherical or polygonal class averages were selected for the 3D structure calculation. Examples of images and averages of undistorted shells can be seen in Roseman *et al.* (2005)[12].

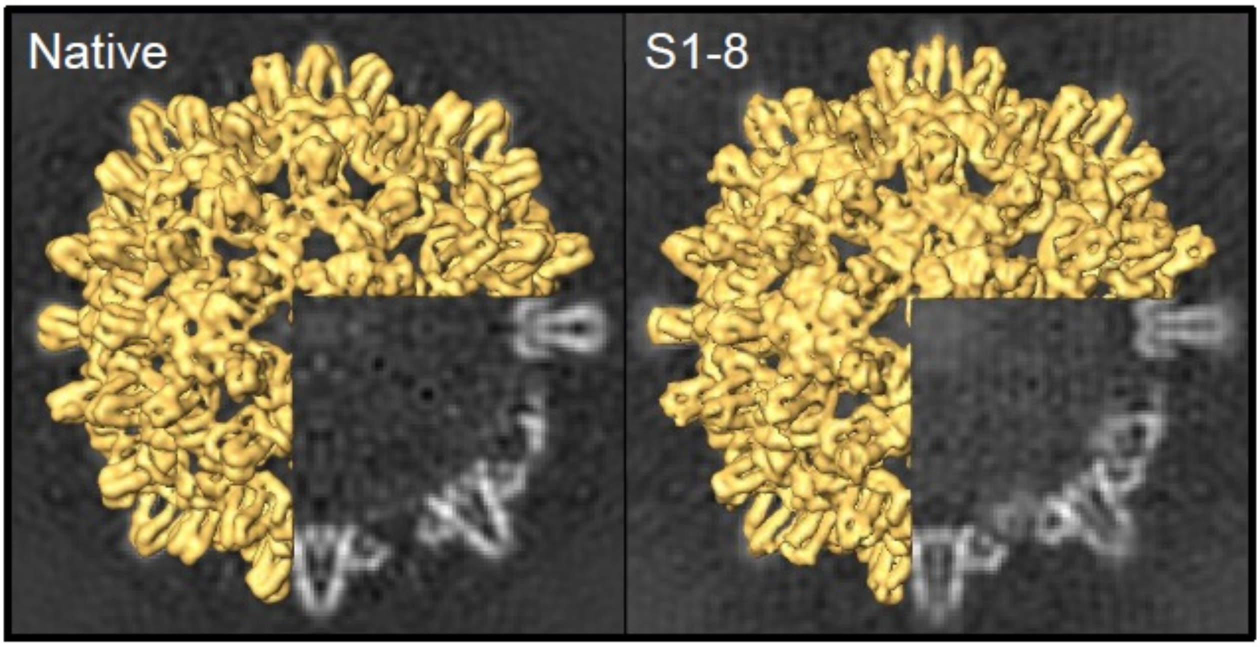

Figure S2. Cross sections of the native and S1-8, T=3 core maps. The 3D maps with a sector cut away are overlaid on sections of the density maps. The section of density shows a weak fuzzy density at the tips of the spikes of the S1-8 core particle, which is not apparent in the native map. This indicates the presence of some disordered material in this construct. The weak density is not apparent in the 3D isosurface representations, as it is below the isosurface threshold. The maps shown are Fourier filtered to 8 Å resolution.

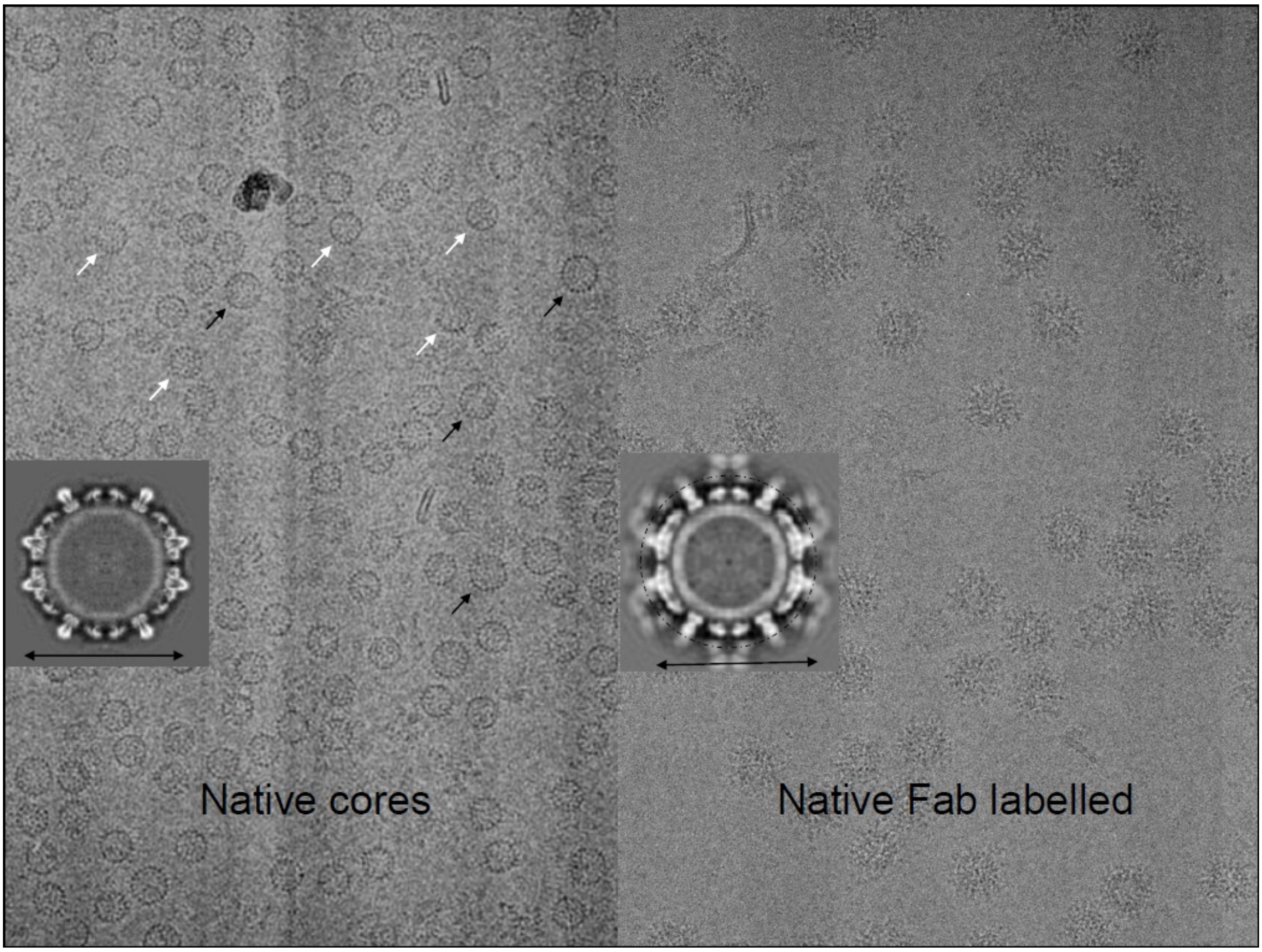

Figure S3. Unlabelled (left) and Fab labelled (right) native core shells, with inset central sections from the 3D maps. In the left panel of unlabelled cores black and white arrows indicate some T=4 shells and T=3 shells respectively. These cores are from the full length sequence, and the weaker inner shell is packaged RNA[12]. The scale bars on the inset map sections indicate the diameter of the unlabelled T=4 core shell, 35 nm. The dotted circle inscribed on the section of the Fab labelled shells indicates the limit of the extent of the inner HBV core shell. The weaker densities outside this boundary are due to the Fabs.

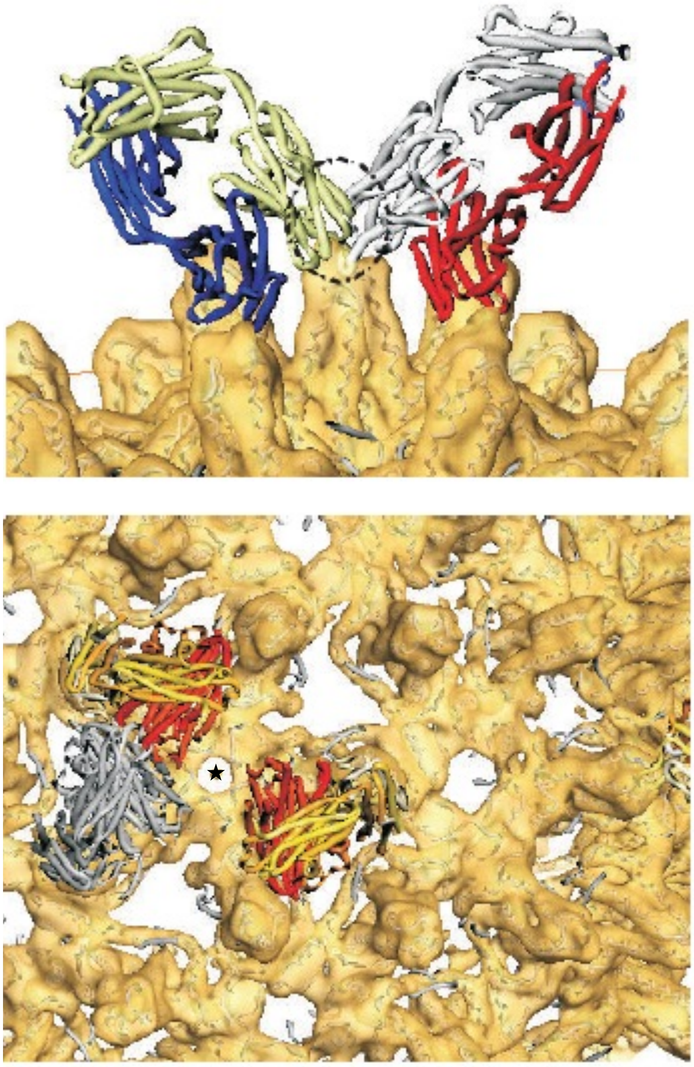

Figure S4. Modelling of blocking of antibody binding sites. The map of the core shell shown is the 8 Å resolution T=4 core shell that was labelled with monoclonal antibody C1-5. The isosurface is set so that the relatively weaker antibody density is not shown (as in Fig. 5b). Top. Two antibodies are positioned on a spike to illustrate the clash preventing two antibodies binding on one spike. The antibody structures are coloured by chain (PDB code 1bbd). Bottom. Two Fabs are shown bound at two opposite sites of the 5 possible symmetry related A-sites that are located around the 5-fold axis (★). These are coloured red and yellow, by chain. It is evident that there is not space for an additional antibody to bind. The additional Fab shown with grey chains clashes with the adjacent red chain. The measured occupancy of C1-5 antibody binding to the native core shell is 0.4, in agreement with the binding or labelling, on average, of two of the five available sites. The fit was determined by docking, as per Fig. 5d, using the antibody model from PDB code 1dba.

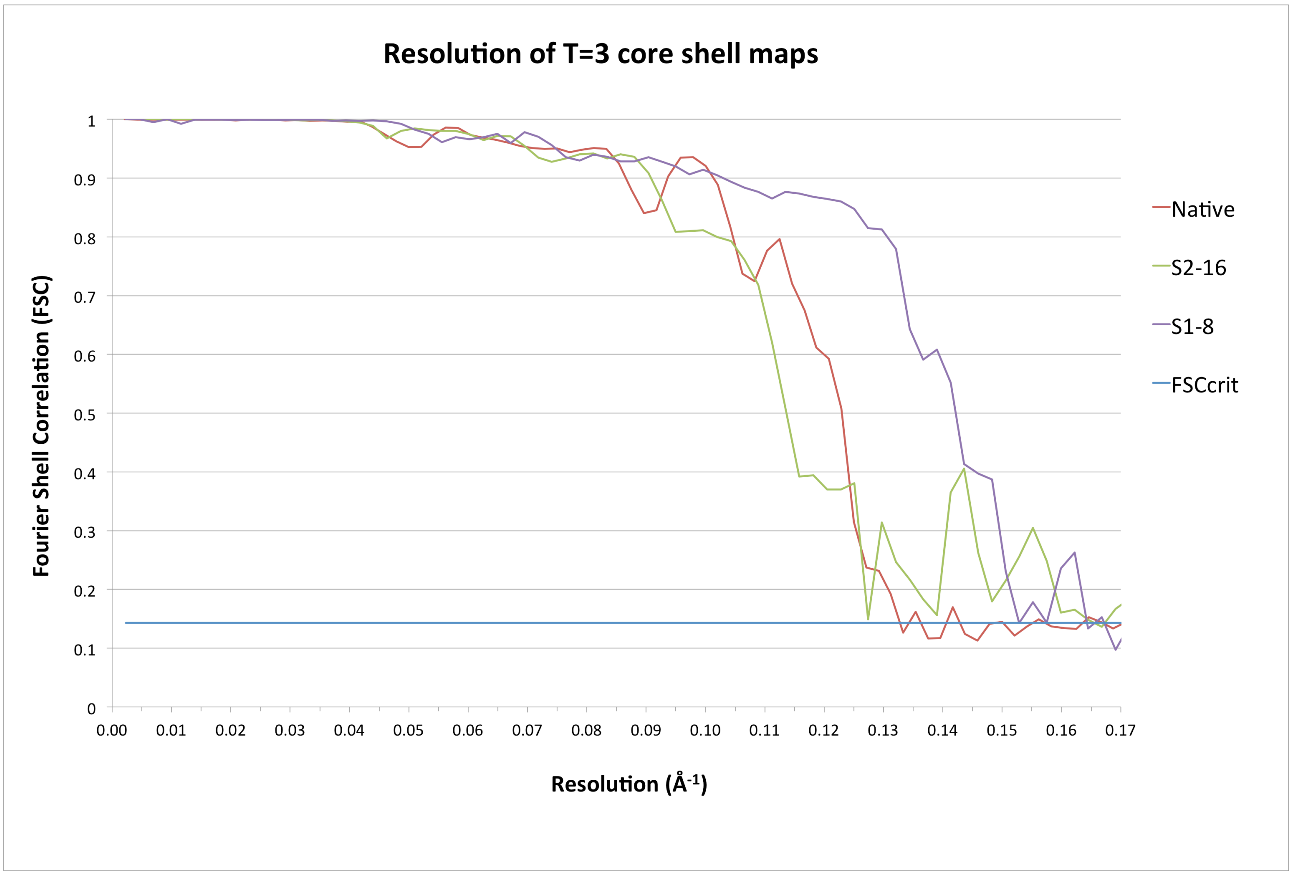

Figure S5. Resolution of T=3 core shell maps. Fourier shell correlations (FSC) were computed from 2 maps made from half the data, for each structure. The map of the native T=3 has a FSC of 0.5 at 8.1 Å (a conservative resolution limit, as used in[17]) and contains information to the limit of 7.6 Å at FSC=0.143 (limit suggested by Rosenthal & Henderson, 2003[52], shown as FSCcrit on the graph). When the FSC computed between the two half maps is above 0.33, then the signal-to-noise ratio in the full map will be better than 1[52]. Both construct maps, S2-16 and S1-8, have a FSC > 0.33 out to past 8 Å resolution. The resolutions at FSC = 0.5 are 8.8 Å and 7.0 Å respectively. In order to define a common resolution at which to be able to compare the maps closely, the criteria of a SNR of 1 was chosen, and these maps were all filtered to 8 Å resolution for comparison with each other.

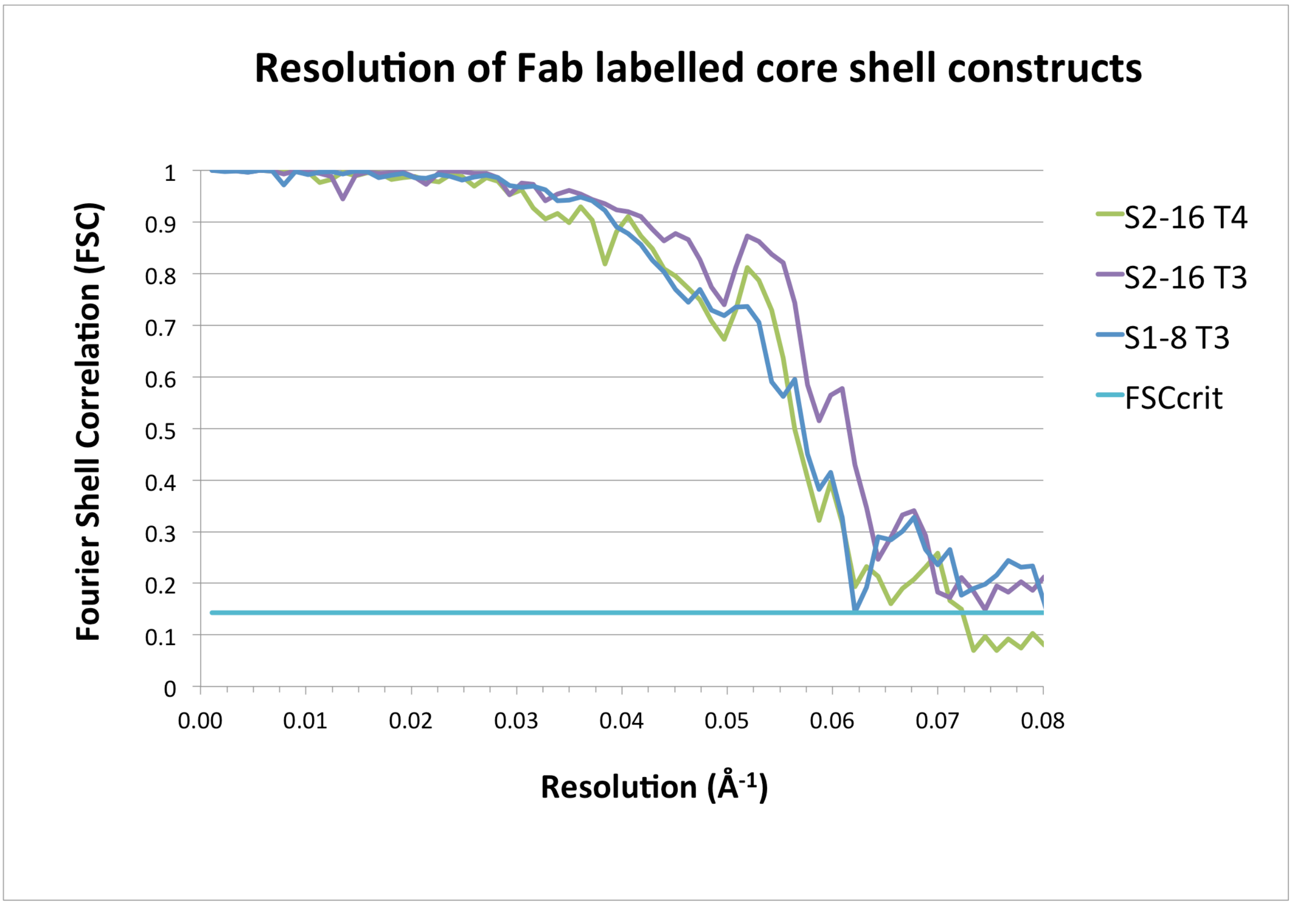

Figure S6. Resolution of Fab labelled T=3 core shells. The FSC between two maps computed by comparing 2 maps calculated from half the data each, for the Fab labelled T=3 core shells. They are all better than ~17.5 Å at FSC=0.5, and contain significant information to at least 14 Å (FSC=0.143, shown on the graph as FSCcrit). The maps were Fourier filtered to 15 Å so they could be displayed at an equivalent resolution.

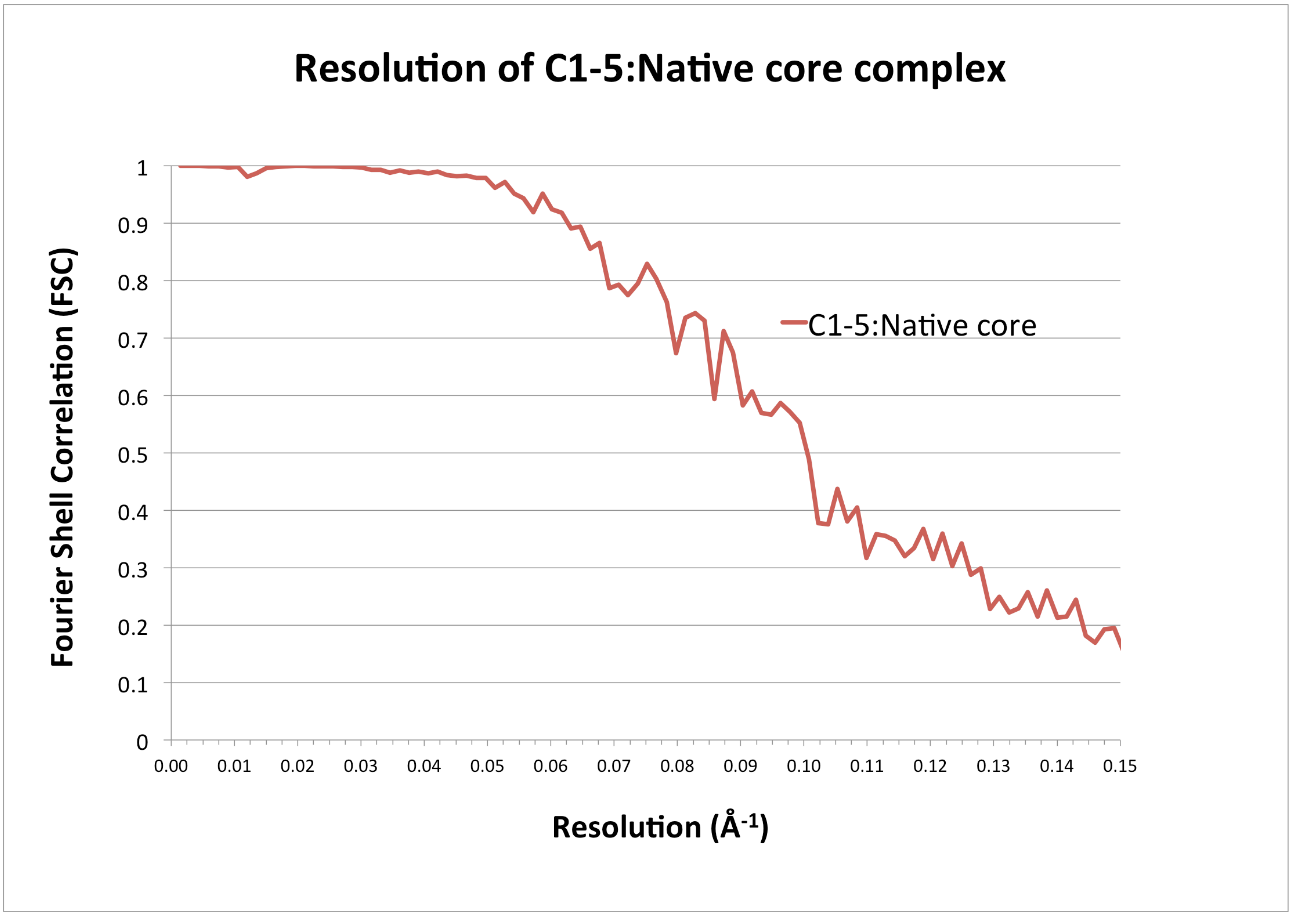

Figure S7. Resolution of the C1-5 Fab labelled T=4 native core shell. The plot shows the FSC calculated from two maps each computed from half the data. The FSC is > 0.5 to better than 10 Å resolution, and remains > 0.33 to 8 Å resolution. The resolution is poorer than the other maps of unlabelled core shells computed from more particles. The partial occupancy of the Fabs will contribute to the lower resolution, as the number of Fabs imaged is lower than core shell subunits.

Map	Films	Particles	Defocus range (µm)
native-T3	13	3740	1.3 – 2.7
native-T4 C1-5	42	6025	1.3 – 4.2
S2-16T3<br>S2-16T3 18/7<br>S2-16T4 18/7	17<br>17<br>12	3305<br>381<br>423	1.5 – 2.9<br>1.8 – 3.7<br>1.9 – 3.9
S1-8T3<br>S1-8T3 18/7	18<br>15	5735<br>256	1.6 – 2.7<br>1.5 – 3.2

Table S1. Imaging parameters for EM map determination of various core shells.
